# In Vitro Characterisation of the Antioxidative Properties of Whey Protein Hydrolysates Generated under pH- and Non pH-Controlled Conditions

**DOI:** 10.3390/foods9050582

**Published:** 2020-05-05

**Authors:** Thanyaporn Kleekayai, Aurélien V. Le Gouic, Barbara Deracinois, Benoit Cudennec, Richard J. FitzGerald

**Affiliations:** 1 Department of Biological Sciences, University of Limerick, V94 T9PX Limerick, Ireland; thanyaporn.kleekayai@ul.ie (T.K.); aurelien.legouic@ul.ie (A.V.L.G.); 2 Joint Research Unit BioEcoAgro N° 1158, Université Lille, INRAE, Université Liège, UPJV, YNCREA, Université d’Artois, Université Littoral Côte d’Opale, ICV Institut Charles Viollette, F-59000 Lille, France; barbara.deracinois@univ-lille.fr (B.D.); benoit.cudennec@univ-lille.fr (B.C.)

**Keywords:** whey protein hydrolysate, hydrolysis condition, food antioxidant, ORAC, cellular ROS, HepG2, peptides

## Abstract

Bovine whey protein concentrate (WPC) was hydrolysed under pH-stat (ST) and non pH-controlled (free-fall, FF) conditions using Debitrase (DBT) and FlavorPro Whey (FPW). The resultant whey protein hydrolysates (WPHs) were assessed for the impact of hydrolysis conditions on the physicochemical and the in vitro antioxidant and intracellular reactive oxygen species (ROS) generation in oxidatively stressed HepG2 cells. Enzyme and hydrolysis condition dependent differences in the physicochemical properties of the hydrolysates were observed, however, the extent of hydrolysis was similar under ST and FF conditions. Significantly higher (*p* < 0.05) in vitro and cellular antioxidant activities were observed for the DBT compared to the FPW–WPHs. The WPHs generated under ST conditions displayed significantly higher (*p* < 0.05) oxygen radical absorbance capacity (ORAC) and Trolox equivalent antioxidant capacity (TEAC) values compared to the FF-WPHs. The impact of hydrolysis conditions was more pronounced in the in vitro compared to the cellular antioxidant assay. WPH peptide profiles (LC-MS/MS) were also enzyme and hydrolysis conditions dependent as illustrated in the case of β-lactoglobulin. Therefore, variation in the profiles of the peptides released may explain the observed differences in the antioxidant activity. Targeted generation of antioxidant hydrolysates needs to consider the hydrolysis conditions and the antioxidant assessment method employed.

## 1. Introduction

Whey is a source of bioactive peptides (BAPs) with a range of biological properties including antihypertensive, antimicrobial, antidiabetic as well as antioxidant activities [1,2]. Consumption of whey protein has been linked with beneficial effects on human health, particularly in the prevention and management of metabolic syndrome conditions such as cardiovascular disease, type II diabetes mellitus, obesity and hypertension [3,4,5]. High intracellular levels of reactive oxygen species (ROS) have been associated with the deleterious modification of cells, nucleic acids (DNA and RNA), proteins and lipids and have also been implicated in accelerating cellular ageing [6]. Cells have different mechanisms to protect themselves from oxidative damage via generation of antioxidant compounds/enzymes, e.g., glutathione, superoxide dismutase (SOD), catalase (CAT) and peroxidase, as well as via the uptake of dietary antioxidants or their precursors [7]. Dietary antioxidants have certain advantages over synthetic antioxidants due to their low risk of side-effects and the fact that they can be included as part of the daily dietary intake [8]. Numerous studies show that whey proteins and their hydrolysates have potential antioxidant effects [1,3,4,5,9,10,11,12,13,14,15,16,17]. Therefore, whey proteins may have potential applications as a source of antioxidant activity in the prevention and management of diseases associated with oxidative stress.

Enzymatic hydrolysis is one of the most effective approaches for liberation of BAPs from intact protein sequences [18]. Due to its mild operating conditions, enzyme-catalysed hydrolysis is extensively used for the generation of food-grade protein hydrolysates. The antioxidant properties of whey protein hydrolysates as well as whey-derived BAPs have been reported to display numerous functions including free radical scavenging, hydrogen and electron donation, metal ion chelation, quenching of singlet oxygen, peroxide decomposition and inhibition of lipid oxidation [3,9,10,12,14,15,16,17,19]. It is well documented that the hydrolysis conditions, e.g., temperature, pH and ionic strength and type of salt influence the characteristics of the hydrolysates obtained [12,20,21,22,23,24,25,26]. The pH of the reaction is considered to be one of the most important parameters during enzymatic hydrolysis. Changes in pH alter the structure of the enzyme as well as its substrate, and consequently can affect enzyme specificity [22,24]. Enzyme specificity determines the resultant peptide profile [24] and, thus, hydrolysate properties [12,20]. The pH can be controlled throughout an hydrolysis reaction by adding acid or base in order to maintain the enzyme at optimum operating conditions. However, this strategy may not be feasible during industrial-scale production. Initially adjusting the pH to the enzyme’s optimum value and then allowing the reaction to proceed uncontrolled is often more feasible during the large-scale production of protein hydrolysates. 

Le Maux et al. [12] demonstrated the impact of hydrolysis under pH- and non pH-controlled conditions on the physicochemical and bioactive properties of whey protein concentrate hydrolysates (WPHs) generated with papain and papain-like proteases. It was shown that the resultant hydrolysates had a similar degree of hydrolysis (DH) but different peptide profiles. This, in turn, led to differences in hydrolysate bioactive properties. For instance, the hydrolysates obtained under pH-controlled conditions had higher oxygen radical absorbance capacity (ORAC) values compared to the non pH-controlled hydrolysis reaction. Similar trends were subsequently reported by Carvalho et al. [20], where a similar DH but different peptide profiles and surface hydrophobicities were observed following whey protein isolate (WPI) hydrolysis using different hydrolysis conditions. 

Generally, protein hydrolysates contain a complex mixture of peptides and amino acids. Therefore, in order to distinguish the antioxidant mechanism(s) of BAPs, it is necessary to employ different antioxidant assays for evaluation of antioxidant potency. Conventionally, assays which measure hydrogen atom transfer (HAT) or electron transfer (ET) are employed in the in vitro assessment of antioxidant activity [27]. For instance, the Trolox equivalent antioxidant capacity (TEAC) assay utilises 2,2’-azino-bis-(3-ethylbenzothiazoline)-6-sulfonic acid (ABTS) and 2,2-diphenyl-1-picrylhydrazyl (DPPH) radicals to measure the HAT and ET activity of test compounds. On the other hand, the ferric reducing antioxidant power (FRAP) is an ET-based assay while the ORAC assay measures the scavenging capacity of test compounds against peroxyl radicals (ROO^•^). The ORAC assay is considered suitable for assessment of the antioxidant activity of protein hydrolysates as it employs biologically relevant radicals [5]. Furthermore, in situ cell-based assays have been recommended as an approach to evaluate antioxidant activity [1]. Cell-based antioxidant assays include assessment of glutathione peroxidase (GPx), CAT and SOD activities, and oxidative damage of DNA, antioxidant gene expression, inhibition of cellular lipid oxidation, protective effects against oxidatively stressed cells and inhibition of cellular ROS generation [3].

The objective of the present study was to investigate the impact of pH- and non pH-controlled enzymatic hydrolysis conditions on the antioxidant properties of WPHs generated using two enzyme preparations. The antioxidant properties of the WPHs were assessed using the in vitro ORAC assay as well as in situ using oxidatively stressed hepatocyte (HepG2) cell lines. In addition, liquid chromatography coupled with mass spectrometry (LC-MS/MS) was employed to identify some of the WPH peptides potentially responsible for the observed antioxidant activity.

## 2. Materials and Methods 

### 2.1. Materials

Whey protein concentrate (WPC80, 80.98% ± 0.68% (*w*/*w*) protein (determined by the Kjeldahl nitrogen determination method)) was obtained from Carbery Group (Balineen, Cork, Ireland). FlavorPro^®^Whey 750P (>55 casein protease U/g) was obtained from Biocatalysts Ltd. (Cefn, Wales, UK) and Debitrase^®^ HYW20 (11,470 U/g) was obtained from DuPont-Danisco (Marlborough, Wiltshire, UK). ABTS, trifluoroacetic acid (TFA), Trolox, 2,2’-azobis (2-amidinopropane) dihydrochloride (AAPH), mass spectrometry (MS) grade water and acetonitrile were purchased from Sigma-Aldrich (Dublin, Ireland). Sodium hydroxide (NaOH) and high pressure liquid chromatography (HPLC) grade water and acetonitrile were provided by Fisher Scientific (Dublin, Ireland). 2,4,6-Trinitrobenzenesulfonic acid (TNBS) was obtained from Pierce Biotechnology (Medical Supply, Dublin, Ireland). Dulbecco’s minimum essential medium (DMEM), fetal bovine serum (FBS), antibiotic-antimycotic solution, L-glutamine, Dulbecco’s phosphate buffered saline (PBS), Hank’s balanced salt solution (HBSS), 2’,7’-dichlorofluorescein-diacetate (DCFH-DA) and dimethyl sulfoxide (DMSO) were purchased from Sigma-Aldrich (Wicklow, Ireland). 

### 2.2. Enzymatic Hydrolysis of WPC80

A 10% (*w*/*v*) protein solution of WPC80 was prepared by reconstitution in distilled water. The protein suspension was mixed at room temperature (22 ± 2 °C) for 2 h and was then allowed to hydrate overnight (16 h) at 4 °C with gentle agitation. The following day, the protein solution was equilibrated at 50 °C followed by adjustment to pH 7.0 using 2 M NaOH. Hydrolysis was initiated by addition of enzyme at an enzyme to substrate ratio (E:S) of 1.0 and 0.5% (*w*/*w*) for FlavorPro^®^Whey 750P and Debitrase^®^ HYW20, respectively. Hydrolysis was carried out at 50 °C for 4 h under gentle agitation. The hydrolysis reaction carried out under ST conditions (Titrando 843, Tiamo 1.4 Metrohm, Dublin, Ireland) was maintained at pH 7.0 for both enzyme preparations. For non pH-controlled conditions (FF), the pH of the solution was monitored throughout the hydrolysis reaction. Aliquots of the hydrolysates were collected at hourly intervals. The reaction was terminated by heating at 80 °C for 20 min. The hydrolysates were then freeze-dried (FreeZone 18 L, Labconco, Kansas City, MO, USA) and stored at −20 °C prior to further analysis.

### 2.3. Determination of Degree of Hydrolysis (DH)

The extent of hydrolysis was determined in triplicate using the TNBS method, as previously described by Le Maux, et al. [12]. The whey protein hydrolysates (WPHs) and unhydrolysed WPC were diluted with 1% (*w*/*v*) SDS to obtain 0.1% (*w*/*v*) protein/protein equivalent solutions. An aliquot (10 µL) was pre-incubated at 50 °C for 30 min prior to mixing with 160 µL of 0.05% (*w*/*v*) TNBS solution in 0.2125 M sodium phosphate buffer pH 8.2. The absorbance at 350 nm was measured after 1 h of incubation at 50 °C using a microplate reader (BioTek Synergy HT, Waltham, MA, USA). Leucine at different concentrations was used as a standard in order to determine the primary amino group content in the samples. The DH was determined using the following formula:

(1) $$\mathrm{DH}\;\left(\%\right)=\frac{{\mathrm{AN}}_{\mathrm{WPH}}\;{-\;\mathrm{AN}}_{\mathrm{WPC}}}{\mathrm{Npb}}\;\times 100$$

where AN_WPH_ is the amino nitrogen content of the hydrolysate (mg nitrogen/mg protein); AN_WPC_ is the amino nitrogen content of the unhydrolyzed WPC and Npb is the nitrogen content of the peptide bonds in whey protein (123.3 mg/g) [28].

### 2.4. Sodium Dodecyl Sulfate Polyacrylamide Gel Electrophoresis (SDS-PAGE)

SDS-PAGE of the WPH samples was carried out using Mini-PROTEAN^®^ TGX™ Precast Gels with a polyacrylamide gradient of 4–20% (Bio-Rad Laboratories Inc., CA, USA) under reducing conditions as described by O’Loughlin et al. [29]. The denatured samples, containing 25 µg protein/protein equivalent, along with the broad range (6.5–200 kDa) molecular mass standard (Bio-Rad) were separated using a mini Protean II electrophoresis system (Bio-Rad) at 150 V for 1 h.

### 2.5. Liquid Chromatography (LC)

The molecular mass distribution and peptide profiles of the freeze-dried WPH samples were analysed using gel permeation high-performance liquid chromatography (GP-HPLC; Waters, Milford, MA, USA) and reverse-phase ultra-performance liquid chromatography (RP-UPLC, Waters), respectively, as previously described by Spellman, et al. [30]. The detector response was monitored at 214 nm. A calibration curve was prepared from the mean retention times of standard proteins and peptides for analysis of molecular mass distribution profiles from the GP-HPLC chromatograms.

### 2.6. In Vitro Antioxidant Analysis

#### 2.6.1. ORAC Assay

The ORAC assay was performed as described by Le Maux et al. [31]. The WPHs were tested at a final concentration of 0.04 mg/mL. Trolox was used as a positive control at final concentrations ranging from 0.0 to 8.0 μM. The ORAC values were expressed as μmol of Trolox equivalents (TE) per g of freeze-dried sample (FDP, *n* = 3).

#### 2.6.2. TEAC Assay

The TEAC assay measures scavenging activity of the test sample against the ABTS cation radical (ABTS^•+^) as described by Re et al. [32], with some modifications. Samples (10 μL) at a final concentration of 0.04 mg FDP/mL were mixed with the ABTS^•+^ working solution (200 μL) in a 96-well microplate. The ABTS^•+^ was monitored at 734 nm following incubation at 30 °C for 6 min. Trolox was used as a positive control at final concentrations ranging from 0.0 to 50.0 μM. The scavenging activity was reported as μmol TE per g FDP (*n* = 3).

### 2.7. Cellular Antioxidant Assay

#### 2.7.1. Tissue Culture

HepG2 (ECACC 85011430) cells were maintained in DMEM supplemented with 10% (*v*/*v*) heat inactivated FBS, 1% (*v*/*v*) non-essential amino acids, 1% (*v*/*v*) antibiotic-antimycotic solution (100 U/mL penicillin, 100 µg/mL streptomycin, 0.25 µg/mL amphotericin B) and 2 mM L-glutamine. Cells were incubated at 37 °C in a humidified environment with 5% CO_2_. HepG2 cells, at passage number 100–110, were used for the experiments. Cell culture medium were replaced every two days, and cells were sub-cultured at 2–4 day intervals before reaching 85%–90% confluence.

#### 2.7.2. Cell Viability

The HepG2 cells were seeded at 6.0 × 10^4^ cells in 200 µL per well on black 96-well plates (Corning, NY, USA) supplemented with DMEM and incubated for 24 h at 37 °C. The medium (200 µL) was aspirated and the adherent cells were rinsed with PBS. The cells were then treated with the WPHs at final concentrations ranging from 0–12.5 mg/mL, prepared in HBSS, and were incubated at 37 °C for 1 h. Following incubation, the medium containing the test compounds was removed and rinsed with PBS. Cell viability was evaluated by exposure to 10% (*v*/*v*) PrestoBlue^®^ (Invitrogen, Biosciences, Dublin, Ireland) in DMEM. The fluorescence of reduced-PrestoBlue due to metabolically active cells was then measured at excitation and emission wavelengths of 560 and 635 nm, respectively, using a microplate reader (Biotek) every 10 min for 2 h at 37 °C. Control cells without treatment with WPH samples were also exposed to PrestoBlue^®^. The analysis was performed in triplicate (*n* = 3) and the results were reported as the percentage of viable cells in the population treated with different WPH concentrations compared to control cells without treatment.

#### 2.7.3. Assay of Intracellular ROS Generation

The cellular antioxidant assay determined the formation of ROS using the oxidation sensitive dye, DCFH-DA, according to the method of Yarnpakdee et al. [33] with some modifications. DCFH-DA was initially prepared at 4 mM in DMSO and was then diluted to 100 µM in HBSS immediately prior to use. The HepG2 cells were seeded at a density of 6.0 × 10^4^ cells in 200 µL per well in black 96-well plates and were then incubated at 37 °C in 5% CO_2_ for 24 h. The medium (200 µL) was aspirated and the adherent cells were rinsed with HBSS. DCFH-DA (100 μL) was added to the cells and the plates were incubated at 37 °C, 5% CO_2_ for 30 min. The cells were treated with the test samples (100 µL) at concentrations ranging from 0 to 10 mg/mL (final concentration) and incubated for 1 h. A positive control containing Trolox at final concentrations of 50 and 100 μM instead of WPH was carried out under the same conditions. An aliquot (100 µL) of medium containing test compounds was removed and 100 µL of 800 µM AAPH in HBSS was added. The fluorescence (excitation: 485 nm, emission: 535 nm) of the 2’,7’-dichlorofluorescein (DCF) product resulting from the oxidation of DCFH in the presence of ROS was measured using a plate reader (Biotek) every 10 min for 90 min at 37 °C. Negative control wells consisted of cells in the presence of DCFH-DA and AAPH without hydrolysates. The intracellular ROS level obtained in the presence of the WPH test samples was expressed as a percentage of the relative fluorescence intensity of the negative control cells.

### 2.8. Peptide Identification by LC-MS/MS

Peptide identification was performed in the 4 h hydrolysates using LC-MS/MS as described by Nongonierma et al. [34]. This consisted of an UltiMate 3000 ultra-HPLC (UHPLC) system (Dionex, Camberley, Surrey, UK) coupled with a quadrupole time-of-flight mass spectrometer (Q-TOF, Impact HD™, Bruker Daltonics GmbH, Bremen, Germany) fitted with an electro-spray ionisation (ESI) source operated in positive ion mode. UHPLC peptide separation was performed using an Aeris Peptide XB-C18 column (150 × 2.1 mm, 1.7 μm; Phenomenex, Cheshire, UK) fitted with a security guard UHPLC C18-PEPTIDE. Mass spectra were scanned at acquisition ranges between 50–600 and 100–2500 m/z for short and long peptides, respectively. Data acquisition was performed using Hystar software (Bruker Daltonics) [35].

Peptide identification was performed using PEAKS Studio (version 7.5, Bioinformatics Solutions Inc., Waterloo, Canada) software and its database search tools. The database used was UniProt_SwissProt (http://www.uniprot.org), taxa *Bos taurus*. The false discovery rate (FDR), average local confidence (ALC) and MS/MS tolerance were set at 1%, 90% and 0.3 Da, respectively. The number of unique and common peptides identified in all samples were subsequently presented in Venn diagram format using the InteractiVenn web-based tool [36]. In addition, peptide abundance was visualised in the form of heat map. Briefly, the occurrence of amino acids within the peptides identified specifically originating from β-lactoglobulin (β-lg) were summated. The results were expressed using a colour code where high, low and no occurrence of an individual amino acid were represented in red, yellow and white, respectively.

Statistical analysis of the peptide maps generated from the LC-MS/MS data acquired from the long peptide detection method was performed using Progenesis QI software for proteomics (Version 4.0, Waters, Milford, MA). The data was subjected to successive processing as follows: (i) alignment of the peptide maps, (ii) peak picking with an intensity threshold set at 2000 and a maximum charge set at 6 and (iii) data standardisation in order to perform statistical analysis on the main components using principle component analysis (PCA). The variables used were derived from the comparison of peptide maps, i.e., the position of the isotopic mass and its intensity.

The peptides identified in the WPHs were searched against the current literature as well as by using the BioPEP-UWM (http://www.uwm.edu.pl/biochemia/index.php/en/biopep) and PepBank (http://pepbank.mgh.harvard.edu/) databases for the presence of previously reported bioactive properties. The location of the identified peptides within the mature bovine β-lg sequence was obtained from Protein BLAST on the National Center for Biotechnology Information (NCBI) resource portal (https://blast.ncbi.nlm.nih.gov/Blast.cgi).

### 2.9. Statistical Analysis

Statistical analysis was performed using IBM SPSS Statistics 24 (IBM, Chicago, IL, USA). The results were analysed by one-way analysis of variance (ANOVA) or student t-test at a significance level of *p* < 0.05. Where applicable, multiple comparisons were performed using Tukey’s post-hoc test.

## 3. Results and Discussion

### 3.1. Degree of Hydrolysis (DH) of WPHs

**Figure 1:**
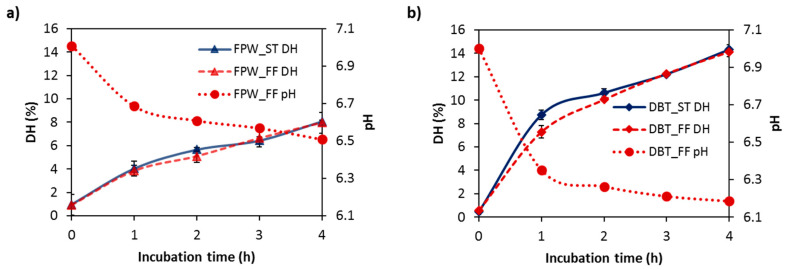
Degree of hydrolysis (DH) and pH profiles as a function of incubation time at 50 °C during the hydrolysis of whey protein concentrate using (**a**) FlavorPro Whey (FPW) and (**b**) Debitrase (DBT) under pH- and non pH-controlled conditions (ST and FF, respectively).

The DH’s achieved as a function of incubation time for the WPHs generated using FlavorPro Whey (FPW) and Debitrase (DBT) under pH- and non pH-controlled conditions are shown in Figure 1. DH increased with incubation time for both enzyme preparations. No major impact of the hydrolysis conditions (ST vs. FF) on the extent of hydrolysis was evident. In general, the DBT–WPHs had a higher extent of hydrolysis compared to the FPW–WPHs with DH values of ~14% and 8%, respectively, being reached following 4 h incubation. The pH of the hydrolysate solutions during FF conditions decreased to ~pH 6.7 and 6.2 for the WPHs generated using FPW (Figure 1a) and DBT (Figure 1b). This decrease in pH during FF hydrolysis is due to the release protons (H^+^) during the cleavage of peptide bonds. Similar DH values for WPHs generated under ST and FF conditions have been reported previously by Le Maux et al. [12] and Carvalho et al. [20].

Both enzyme preparations used during WPC hydrolysis contain microbial proteinase activities enriched with exopeptidase activity. FlavorPro Whey 750P derived from *Aspergillus spp.* is reported to cleave at L, F, K, M, E, V, T and C residues (Biocatalyst Technical Bulletin Revision 2: 24 September 2014). Debitrase HYW20 derived from *Aspergillus oryzae* and *Bacillus spp.* possesses leucine aminopeptidase (LAP) and post-proline dipeptidyl aminopeptidase activities [37]. The fact that Debitrase contains proteases from *Bacillus spp.*, which generally have broad specificity [38], may have contributed to the higher extent of hydrolysis observed in the hydrolysates with this enzyme. The application of both enzyme preparations (FPW and DBT) has previously been reported to reduce bitterness in milk protein hydrolysates [39,40].

Analysis of DH only provides an indication of the overall extent of peptide bond cleavage compared to the unhydrolysed sample. However, it does not give any information on the mechanism of hydrolysis or on which peptide bonds were hydrolysed [26]. Therefore, the observation of similar DH values between the ST and FF conditions for each enzyme preparation does not imply similar cleavage sites during WPC hydrolysis. In addition, not all the cleavage sites are hydrolysed at the same rate. This is due to the fact that the rate of hydrolysis of a specific cleavage site is affected by the presence of other amino acids (subsite) in the position adjacent to the cleavage site [24,26,41]. Therefore, further investigation on the impact of hydrolysis conditions on the peptide profile and the antioxidant properties of the WPHs was carried out herein.

### 3.2. Electrophoresis and Molecular Mass Distribution Profiles

**Figure 2:**
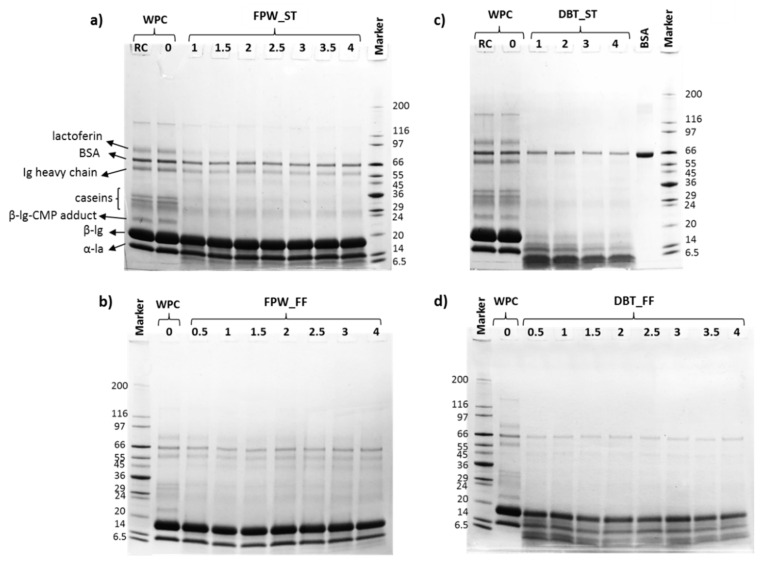
Gel electrophoresis profiles of the reconstituted (RC) intact whey protein concentrate (WPC80), WPC 0 h (WPC0) and the whey protein hydrolysates (WPHs) generated using (**a**) FlavorPro Whey under pH control (FPW_ST), (**b**) FPW without pH control (FPW_FF), (**c**) Debitrase under pH control (DBT_ST) and (**d**) DBT without pH control (DBT_FF) as a function of incubation time (h) at 50 °C. BSA: bovine serum albumin; CMP: caseinomacropeptide; β-lg: β-lactoglobulin; α-la: α-lactalbumin.

The electrophoretic profiles showed the degradation of protein bands corresponding to the major intact whey proteins (β-lg and α-lactalbumin (α-la)), as well as the generation of low molecular mass compounds <6.5 kDa in the WPHs. This degradation was influenced by the enzyme preparation used for hydrolysis and the hydrolysis conditions, as shown in Figure 2. However, it was noted that a band corresponding to bovine serum albumin (BSA) was observed throughout the incubation period for all samples, albeit with lower intensity compared to that in unhydrolysed WPC. The electrophoretic profiles also show that the WPHs generated using FPW displayed a limited extent of hydrolysis of the main whey protein bands which agrees with the lower extent of WPC hydrolysis compared to that observed in the DBT–WPHs (Figure 1 and Figure 2).

In addition, these results highlighted that the WPHs generated under different hydrolysis conditions (ST vs. FF) despite having similar DH values (Figure 1), showed different WPC digestion profiles, particularly in the case of DBT–WPHs (Figure 2c,d). Previous studies by Le Maux et al. [12], Carvalho et al. [20] and Fernández and Kelly [23] also reported different digestion profiles between whey protein hydrolysed under ST and FF conditions (while having comparable DH values). Butré et al. [24] demonstrated significant changes in enzyme selectivity (up to 80%) toward cleavage sites in β-lg as a function of pH which also resulted in different hydrolysate molecular mass distribution profiles. This indicates that the kinetics of peptide release were influenced by the changes in pH during the FF conditions. The changes in enzyme selectivity were previously attributed to modifications in the charge state of amino acids at the active site of the enzyme and at the site of cleavage, as well as in the region adjacent to the cleavage sites [24].

**Figure 3:**
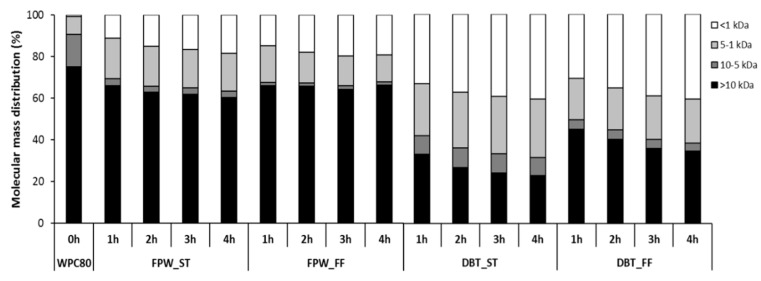
Molecular mass distribution profile of unhydrolysed whey protein concentrate (WPC80) and the whey protein hydrolysates (WPHs) generated using FlavorPro Whey (FPW) and Debitrase (DBT) under pH- and non pH-controlled conditions (ST and FF, respectively) during the course of a 4 h hydrolysis period.

The molecular mass distribution profiles (Figure 3) obtained following GP-HPLC of the WPHs displayed similar results to those observed in the electrophoresis profiles. A greater proportion of high molecular mass components (>10 kDa) was observed in the FF_WPHs compared to the ST_WPHs during hydrolysis with both enzyme preparations. In contrast, Le Maux et al. [12] reported a higher proportion of high molecular mass components (>10 kDa) in ST generated hydrolysates compared to FF conditions for WPC hydrolysates generated with papain. However, in the case of WPHs generated with papain-like activity there was no major differences between the molecular mass profiles of the ST and FF hydrolysates. This indicated that the effect of hydrolysis conditions on the molecular mass distribution profiles was enzyme-dependent. These differences may be explained by the lower optimum pH range of papain, i.e., between pH 5–8, when compared to DBT and FPW which have optimum pH values between pH 6–8.

A general correlation between the molecular mass distribution profiles and DH was evident in that the proportion of peptides <1 kDa increased as a function of incubation time in all the WPH samples. The DBT–WPHs which had higher DHs than the FPW–WPHs had a higher proportion of low (<1 kDa) molecular mass components (Figure 3). The relatively high proportion of high molecular mass components (>10 kDa) in all hydrolysates (ranging between 20% and 70%) may be related to a relatively low level of broad specificity proteinase activities in the FPW and DBT preparations. This may be related to the fact that these enzymes are primarily marketed as exopeptidase containing preparations for protein hydrolysate debittering applications.

### 3.3. Reverse-Phase (RP) Peptide Profiles

**Figure 4:**
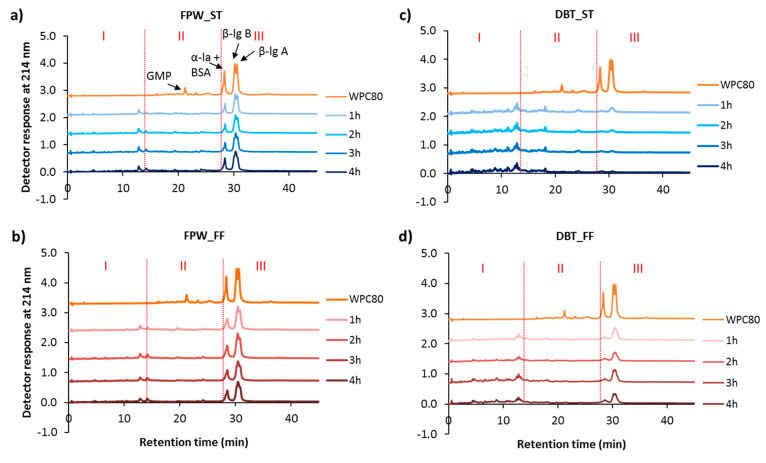
Reverse-phase ultra-performance liquid chromatographic (RP-UPLC) profiles of unhydrolysed whey protein concentrate (WPC80) and the whey protein hydrolysates (WPHs) generated using (**a**) FlavorPro Whey under pH controlled (FPW_ST), (**b**) FlavorPro Whey under non pH controlled (FPW_FF), (**c**) Debitrase under pH controlled (DBT_ST) and (**d**) Debitrase under non pH controlled (DBT_FF) conditions during the course of 4 h hydrolysis. Regions labelled I, II and III represent linear acetonitrile gradients between 0–20%, 20%–40% and >40%, respectively. β-lg: β-lactoglobulin; α-la: α-lactalbumin; BSA: bovine serum albumin; GMP: glycomacropeptide.

The peptide profiles of the hydrolysates generated are shown in Figure 4. The degradation of the intact proteins in the WPC as well as the release of hydrophilic peptides was observed with both enzyme preparations, however, this was more pronounced in the case of DBT–WPHs. In addition, the hydrolytic enzymes had a major impact on the peptide profiles of the WPHs with a greater extent of hydrolysis of the intact whey proteins being observed in the DBT–WPHs (Figure 4c,d). Furthermore, the DBT_ST contained a limited amount of intact whey proteins while the DBT_FF hydrolysates had some remaining intact β-lg (Figure 4d). The influence of hydrolysis conditions (ST vs. FF) on peptide profiles concurs with previous reports by Le Maux et al. [12], Butré et al. [24] and Carvalho et al. [20]. Butré et al. [24] showed that, at similar DH, WPI hydrolysed under different constant pH values (pH 7.0–9.0) resulted in different concentrations of residual intact proteins, including β-lg, in the hydrolysates. Le Maux et al. [12] reported that WPC hydrolysed using papain or papain-like activity with ST and FF conditions had comparable overall peptide profiles with different intensities in some peptide peaks. In addition, Fernández and Kelly [23] suggested that different hydrolysis conditions resulted in different kinetics of peptide release, where a slower reaction rate occurred in the FF in comparison to the ST conditions. Carvalho et al. [20] demonstrated that whey protein hydrolysates generated without pH control exhibited significantly higher surface hydrophobicities than those produced under pH control. Therefore, the change in pH during FF hydrolysis may lead to changes in enzyme cleavage specificity resulting in different peptide profiles being observed in comparison to those of the hydrolysates generated under ST.

### 3.4. In Vitro Antioxidant Properties

**Figure 5:**
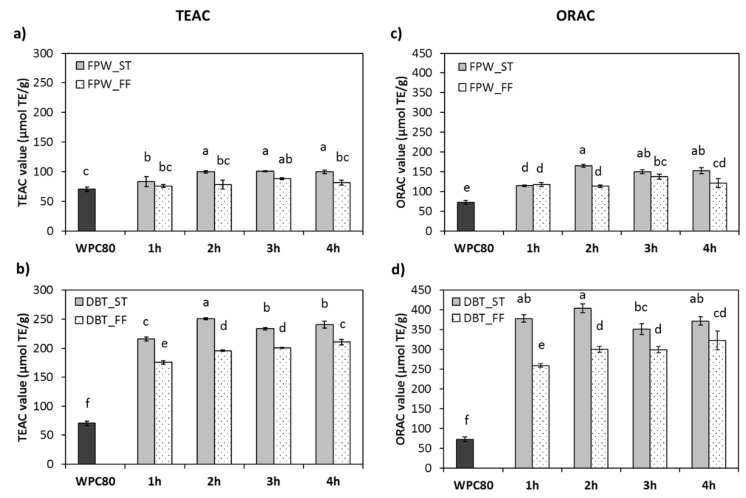
In vitro antioxidant activities reported as Trolox equivalent antioxidant capacity (TEAC) and oxygen radical absorbance capacity (ORAC) of unhydrolysed whey protein concentrate (WPC80) and the whey protein hydrolysates (WPHs) generated using (**a**) and (**c**) FlavorPro Whey under pH- and non pH-controlled (FPW_ST and FPW_FF, respectively) and (**b**) and (**d**) Debitrase under pH- and non pH-controlled (DBT_ST and DBT_FF, respectively) conditions during the course of 4 h hydrolysis period. Values reported are mean ± SD, *n* = 3. Different letters indicated significant difference at *p* < 0.05.

The in vitro antioxidant properties of the WPHs generated with FPW and DBT under ST and FF conditions were assessed using the TEAC and ORAC assays and the results are shown in Figure 5a–d. The TEAC and ORAC values of the WPHs were in the range of 76.0–250.6 and 113.3–403.9 µmol TE/g, respectively, depending on the enzyme and hydrolysis conditions used. In general, the antioxidant properties of all hydrolysates were significantly higher than unhydrolysed WPC with the exception of the TEAC values for the FPW_FF WPHs (Figure 5). These results were generally comparable to the previously reported by other studies. Le Maux et al. [31] reported ORAC values ranging from 179.5–227.6 µmol TE/g for Protamax-WPHs. Power et al. [42] reported an ORAC value for a β-lg tryptic hydrolysate of 467.65 µmol TE/g dry weight.

No major further changes in the antioxidant activities of all hydrolysates occurred after 1 h incubation with the enzymes (Figure 5). This may be due to the higher rate of hydrolysis in the first hour of incubation (as shown in Figure 1), for both enzyme preparations. These results were confirmed by the molecular mass distribution and the peptide profiles (Figure 3 and Figure 4). The lower antioxidant activities in the FPW–WPHs may be associated with the lower extent of hydrolysis in these samples. This finding indicates that the WPHs generated by DBT had more efficient hydrogen atom and electron transfer abilities (ABTS assay) as well as scavenging activity against peroxyl radicals (ORAC assay) than the FPW–WPHs. A number of previous studies also demonstrated an enzyme-dependent effect on the in vitro antioxidant potencies of WPHs. Mann et al. [43] reported the antioxidant activity of WPHs generate using Flavourzyme, Alcalase or Corolase PP having TEAC values ranging from 0.81–1.42 µM TE/mg protein. They also suggested that the TEAC values of the WPHs were associated with the extent of hydrolysis which was also in agreement with the results of Salami et al. [17] when hydrolysing whey proteins using Proteinase K, thermolysin, trypsin or chymotrypsin. A significant increase in DPPH scavenging activity as a function of incubation time was observed in the WPH generated using a serine protease from *Myceliophthora thermophile*, while ferric chelation activity did not change after 3 h incubation (*p* > 0.05). No significant change in antioxidant properties was observed in the case of WPH hydrolysed with a metalloprotease from *Eupenicillium javanicum* [14]. These results therefore indicated that the antioxidant properties of WPHs are influenced by the enzyme preparation used which may in turn be linked to the specificity and the extent of hydrolysis achieved. However, O’Keeffe and FitzGerald [15] reported that the ORAC values of a 5 kDa permeate fraction of WPC hydrolysed with Alcalase, Neutrase, Corolase PP or Flavourzyme were not significantly (*p* > 0.05) different (0.6–0.9 µmol TE/mg sample), while the DH ranged between 11.4%–20.5%.

The results showed that the ST conditions resulted in significantly higher antioxidant activities compared to the FF conditions for both enzyme preparations following 4 h incubation (Figure 5). This may be linked to the different physicochemical characteristics, i.e., electrophoretic, molecular mass distribution and reverse-phase profiles, of the ST- vs. FF-WPHs generated using the same enzyme preparation (Figure 2, Figure 3 and Figure 4). Le Maux et al. [12] reported ORAC values for WPHs ranging from 193–308 µmol TE/g depending on the enzyme and the hydrolysis condition used. The highest ORAC value was found in the WPHs generated under ST (pH 7.0) conditions which was significantly higher than for the WPHs generated at either a lower constant pH (pH < 7.0) or during FF conditions (*p* < 0.05). Therefore, these results showed that controlling the pH at enzyme optimum values can contribute to the release of more potent antioxidant peptides at least when hydrolysing WPC with FPW and DBT.

### 3.5. Cellular Antioxidant Activity

Biochemical antioxidant assays are considered as generic in vitro assays where the results obtained may not be readily translated to more complex systems such as in the human body [43]. Therefore, it is useful to assess antioxidant properties using in situ cellular-based assays which may be more representative of the target site of oxidative stress in vivo. Several studies reported on the application of cellular antioxidant-based assays of whey protein and its derivatives using various cell lines, e.g., mice myoblast (C2C12) [44,45], human lung fibroblast (MRC-5) [46], rat pheochromocytoma (PC12) [47], human colonic adenocarcinoma (Caco-2) [48], human umbilical vein endothelial (HUVECs) [15] and human tracheobronchial epithelial (1HAEo) [49] as well as human hepatocyte (HepG2) cells [45,50], as reviewed by Corrochano et al. [3]. 

The cell cytotoxicity of two oxidative stress inducers (AAPH and H_2_O_2_) was pre-evaluated herein at concentrations ranging from 0–1,000 µM in order to investigate their potential toxic effects on HepG2 cells. The results showed that both inducers resulted in similar effects on cell viability (Supplementary Data, Figure S1). A toxic effect yielding <70% cell viability was found at levels >700 µM for both type of inducer. Due to the similar effects observed between AAPH and H_2_O_2_, AAPH was selected to represent the oxidative stress inducer at a concentration of 800 µM giving cell viability at a minimum of 50%. In addition, peroxyl radicals from AAPH are reported to have a longer half-life than hydroxyl radicals generated from H_2_O_2_ at 10^−2^ and 10^−9^ s, respectively [51]. Furthermore, the cell viability using PrestoBlue^®^ of the 4 h-WPHs was evaluated at different concentrations up to 12.5 mg/mL. The WPHs did not appear to have an impact on cell viability with >99% viability (Supplementary Data, Figure S2). Therefore, 3 concentrations of the WPHs, i.e., 1, 5 and 10 mg/mL (final concentrations), were selected for further investigation on their cellular antioxidant activity effects.

**Figure 6:**
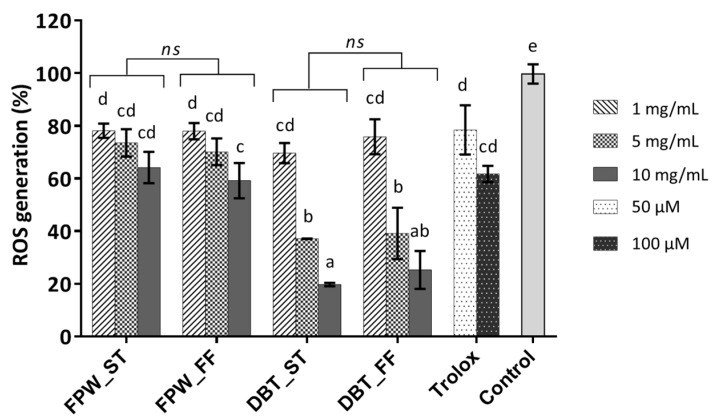
Extent of intracellular reactive oxygen species (ROS) generation in 2,2’-azobis (2-amidinopropane) dihydrochloride (AAPH) stressed-HepG2 cells treated with 1–10 mg/mL (final concentration) of the 4 h whey protein hydrolysates generated using FlavorPro Whey (FPW) and Debitrase (DBT) under pH- (ST) and non pH-controlled (FF) conditions. Trolox at 50 and 100 µM was used as a positive control. Values reported are mean ± SD, *n* = 3. Different letters indicated significant difference at *p* < 0.05. ns: non-significant (*p* ≥ 0.05).

The cellular antioxidant assay was carried out to assess the reduction effect of the test samples against AAPH induced intracellular ROS generation, as per Yarnpakdee et al. [33]. The commercial antioxidant, Trolox, was used as a positive control. As expected, Trolox led to a significant reduction in ROS generation compared to the negative control, i.e., AAPH-stressed cells, which was considered to yield 100% ROS generation (Figure 6). Treatment of the cells with 50 or 100 µM Trolox did not show significant differences in the reduction of ROS generation (with 81.09% ± 9.26% and 61.65% ± 4.06%, respectively). Intracellular ROS generation in the WPHs treated cells was in the range 20%–78%. The result therefore showed that treatment with the WPHs led to lower levels of ROS generation. The FPW–WPHs had limited effect on ROS generation (59.2%–78.2%) compared to the DBT–WPHs which showed a greater range in the reduction of ROS generation (19.7%–75.9%).

The DBT_ST WPH at 10 mg/mL exhibited the most potent cellular ROS generation reducing activity giving an ~80% reduction in intracellular ROS generation in AAPH-stressed HepG2 cells in comparison to the control. Honzel et al. [52] associated such a strong inhibition of cellular ROS formation with anti-inflammatory properties. A WPI hydrolysate generated with the aid of high pressure pre-treatment (at 550 MPa) was reported to inhibit the effects of the pro-inflammatory cytokine (IL-8) and ROS generation by up to 50% and 76%, respectively, in H_2_O_2_-stressed Caco-2 cells in a dose-dependent manner [48]. Likewise, Bamdad et al. [9] reported on high hydrostatic pressure assisted β-lg hydrolysates (BLGHs) displaying an improvement in antioxidant activity and anti-inflammatory properties in lipopolysaccharide (LPS)-stimulated RAW264.7 macrophage cells. The antioxidant activity of the BLGHs was also enzyme-dependent. In the case of anti-inflammatory properties, the BLGHs reduced the nitric oxide level and showed suppression of pro-inflammatory cytokines (tumor necrosis factor (TNF-α) and IL-1β) in LPS-stimulated RAW264.7 cells.

With the exception of DBT-FF WPH at 5 and 10 mg/mL, the reduction of intracellular ROS generation of the DBT–WPHs treated cells was observed to occur in a dose-dependent manner (*p* < 0.05). The greater reduction in ROS generation in the HepG2 cells treated with the DBT–WPHs concurred with the results observed in the in vitro TEAC and ORAC assays (Figure 5b and d, respectively). This may be associated with the higher extent of hydrolysis in these samples (Figure 1). The hydrolysis conditions (ST vs. FF) did not have a major impact on intracellular ROS generation for the hydrolysates generated with either DBT or FPW (Figure 6). To our knowledge, this is the first report demonstrating the contribution of hydrolysis conditions on the cellular antioxidant activity of WPHs.

Nonetheless, Honzel et al. [52] indicated that the magnitude of reduction in intracellular ROS generation was not directly correlated with ORAC assay values. In addition, the cell-based antioxidant activity also depends on the permeability of the test sample [53] as well as the interaction between the test sample and complex enzyme reactions in biological systems [52]. Kong, et al. [46] reported that WPHs enhanced SOD, GPx and CAT activities in H_2_O_2_-induced MRC-5 cells. Similar results were also observed by O’Keeffe and FitzGerald [15], where WPHs obtained following membrane filtration (5 kDa) resulted in an increase in the expression of glutathione and CAT activity in HUVECs. On the other hand, the level of antioxidative biomarkers, i.e., glutathione pyruvate transaminase, alkaline phosphatase and creatinine in HepG2 cells decreased in the presence of WPHs [54]. The 3 kDa permeate fraction of a peptic-digest of whey protein derived from buffalo colostrum restored the level of ROS, H_2_O_2_ and CAT to normal. In addition, it replenished the glutathione level and moderately restored lysosomal enzyme activity in 2,4-ditrophenol (DNP)-induced oxidatively stressed human blood samples [55].

### 3.6. Peptide Identification by LC-MS/MS

**Figure 7:**
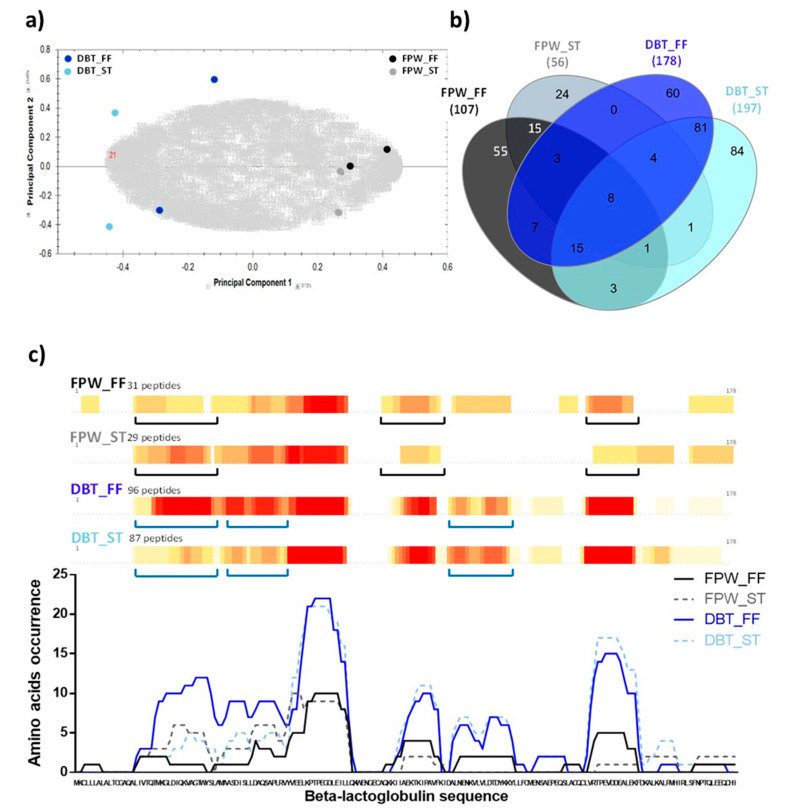
Overall number of common and unique peptides identified in the 4 h whey protein hydrolysates generated using FlavorPro Whey (FPW) and Debitrase (DBT) under pH- and non pH-controlled conditions (ST and FF, respectively). (**a**) Principal component analysis based on comparison of mass spectrometry detected peptides in the WPHs, (**b**) Venn diagram representing the overall number of identified peptides in the WPHs with the common peptide identified in several samples represented by the overlapping area in the diagram and (**c**) Heat map showing amino acid occurrence in the identified peptides in the primary sequence of β-lactoglobulin where the red colour represents high number of identifications, yellow represents low number of identifications and white represents unidentified amino acids. Underlined regions highlight peptide pattern differences between ST and FF conditions for FPW (in black) or DBT (in blue) hydrolysis.

In order to investigate the hydrolysis pattern and enzyme specificities in the FPW/DBT_ST and FPW/DBT_FF hydrolysates, the digests obtained following 4 h incubation were selected for peptide identification by LC-MS/MS. PCA was performed on the mass spectrometry data, more particularly on the detected ions (Figure 7a). The first two dimensions in the PCA explained 61.37% of the variance (detected ions corresponding to variables). The more distant the groups were, the more different they were in terms of ion population. The PCA clearly showed a different ion population and thus a different peptide population between the FPW and DBT hydrolysates.

The total number of peptides identified in the 4 h FPW_FF, FPW_ST, DBT_FF and DBT_ST WPH samples was 107, 56, 178 and 197, respectively. The common and unique peptides in all the samples were identified and the results are presented in Venn diagram format in Figure 7b. This analysis showed that the 4 WPHs, which were derived from two different enzyme preparations and two different hydrolysis conditions, contained eight common peptides. Each hydrolysate sample also had unique peptides, i.e., 55, 24, 60 and 84 peptides in FPW_FF, FPW_ST, DBT_FF and DBT_ST, respectively, (Figure 7b). The higher number of peptides identified in the DBT-WPH samples may be linked to the higher DHs in these samples compared to the FPW–WPHs (Figure 1). However, no clear pattern could be observed in the Venn diagram concerning the effect of hydrolysis conditions on the peptides released. Therefore, the hydrolysis pattern of the major intact whey protein, β-lg, was assessed and the results are presented using a heat map diagram as shown in Figure 7c. This analysis clearly showed that the hydrolysis conditions, i.e., ST vs. FF for the same enzyme preparation, resulted in different cleavage patterns on β-lg (Figure 7c and Table 1). These results are in agreement with those previously reported by Butré et al. [24] on the effect of hydrolysis conditions on peptide profile. Furthermore, the occurrence of specific amino acids in the peptides released was hydrolysis condition-dependent, as illustrated in the regions underlined on the heat map (Figure 7c). This finding may help to explain the differences in the observed antioxidant properties of the hydrolysates herein.

The β-lg-derived peptides identified in the hydrolysates with <10 amino acid residues and containing antioxidant peptide features or having related sequences to those which were previously reported to be bioactive are presented in Table 1. The majority of the peptides identified in the FPW–WPHs were long sequences, i.e., with >56% and >75% of all peptides identified having >10 amino acid residues in the FPW_FF and FPW_ST WPHs, respectively (data not shown). This may be associated with the relatively low extent of hydrolysis in these samples (Figure 1). Some of the peptides identified in the WPHs obtained in the present study have been previously reported to possess antioxidant activity. For instance, LDTDYKK (β-lg f(95–101)) was present in WPC enriched in β-lg when hydrolysed with Corolase PP and thermolysin exerted ORAC antioxidant activity [10]. VLDTDYK (β-lg f(94–100)) and VRTPEVDDE (β-lg f(123–131)), derived from Alcalase hydrolysed cheese whey, had ABTS^•+^ scavenging activity [56]. GTWYSL (β-lg f(17–22)), AMAASDISLL (β-lg f(23–32)), MAASDISL (β-lg f(24–32)) and IIAEKTKIPAVF (β-lg f(71–82)) identified in Alcalase hydrolysed β-lg under high hydrostatic pressure also showed ferric reducing antioxidant activity [9]. In addition, TPEVDDEALEK (β-lg f(125–135)) which was identified in all four hydrolysates in the present study (Table 1) was previously found in WPC hydrolysed with Flavourzyme and Corolase PP exerted ABTS^•+^ scavenging activity [57]. The same peptide was reported in a tryptic β-lg hydrolysate and had ORAC activity (0.004 µmol TE/µmol peptide) [42]. Among the β-lg-derived peptides identified in the present samples, three peptides (VLDTDYK, VRTPEVDDEALEK and TPEVDDEALEK) were not only reported to be resistant to in vitro gastrointestinal digestion, but they also had the capability to be transported across the intestinal epithelium (Caco-2 monolayers) [58]. 

**Figure 8:**
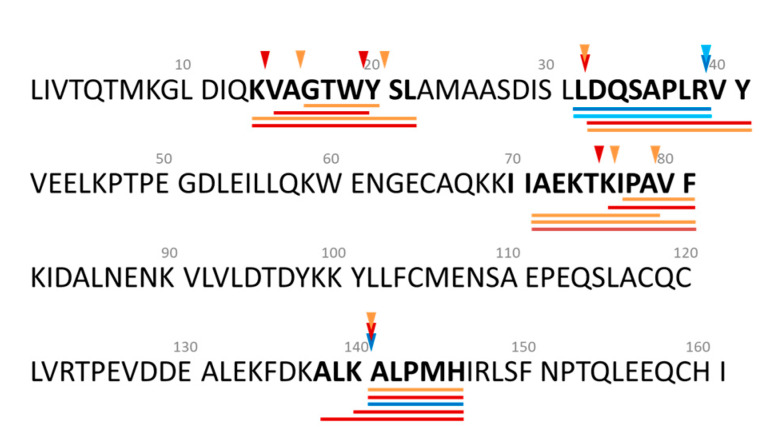
Primary sequence of bovine β-lactoglobulin showing specific cleavage sites and the peptides identified in whey protein concentrate hydrolysed using FlavorPro Whey (FPW) under pH-controlled (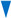), non pH-controlled (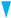) conditions and Debitrase (DBT) under pH-controlled (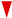) and non pH-controlled (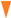) conditions. Peptide sequences underlined in different colours indicate the peptides identified in the samples described above.

In general, enzyme preparation plays a key role in peptide release during enzymatic hydrolysis of food proteins and consequently influences hydrolysate bioactive potency. In the present study, two enzyme preparations were used to hydrolyse WPC which led to different profiles of peptides released. For example, LDAQSAPLR (β-lg f(32–40)) and DAQSAPLRVY (β-lg f(33–42)) which were identified in FPW_ST and FF and in DBT_ST and FF, respectively (Table 1), clearly illustrates the differences in the cleavage specificities between the two enzyme preparations. Cleavage post Leu occurred in the case of DBT yielding f(33−42). This may be linked to the presence of LAP in *A. oryzae* [37], while FPW cleaved post Arg in this region of the β-lg molecule (Figure 8).

Apart from the enzyme preparation used, the effect of hydrolysis conditions, ST vs. FF, for the same enzyme preparation was evident from the different peptides released from specific β-lg regions, e.g., f(15−22), f(73−82) and f(139−146) as shown in Figure 8. AEKTKIPAVF (β-lg f(73−82)) was present in DBT_FF and ST and appeared to act as an intermediate sequence for further hydrolysis by DBT. This, in turn, resulted in different peptides being released depending on the hydrolysis conditions, i.e., AEKTKIPA (β-lg f(73−80)) and IPAVF (β-lg f(78−82)) were identified in DBT_FF while KIPAVF (β-lg f(77−82)) was only found in DBT_ST (Table 1). This result indicates that hydrolysis conditions influenced the cleavage specificities for β-lg.

Similar observations can be made in the case of KVAGTWYSL (β-lg f(14−22)) which was detected in both DBT_FF and ST. Its derivatives, i.e., VAGTW (β-lg f(15−19)) and GTWYSL (β-lg f(17−22)), were present in DBT_ST and DBT_FF, respectively. Peptides related to β-lg f(14−22) have been reported to exert antioxidant properties, e.g., VAGTWY (β-lg f(15−20)) and VAGT (β-lg f(15−18)) showed ORAC values of 5.63 µmol TE/µmol peptide [42] and 1.66 µmol TE/mmol peptide [60], respectively. Furthermore, WYSL (β-lg f(19−22)) possessed DPPH and superoxide radical scavenging activity with EC_50_ values of 273.63 and 558.42 µM, respectively [61].

The lactokinin, ALPMHIR (β-lg f(142−148)), have been reported to exerted angiotensin I-converting enzyme (ACE) and dipeptidyl peptidase IV (DPP-IV) inhibitory and insulinotropic activities [59,68,69,70,71]. In addition, it has in vitro antioxidant activity with a reported ORAC value of 0.035 µmol TE/µmol peptide [42]. In the present study, ALPMH (β-lg f(142−146)) was detected in both DBT_ST and FF, whereas ALKALPM (β-lg f(139−145) and KALPM (β-lg f(141−145) were only detected in the DBT_ST WPH. In the case of FPW, only ALPMH was found in the FPW_ST WPH, while no lactokinin fragments (with >90% average local confidence) were detected in FPW_FF. The different peptides released from the WPHs generated using DBT and FPW under ST and FF conditions may explain the differences in antioxidant potencies of the resultant hydrolysates.

## 4. Conclusions

This study demonstrated the presence of antioxidant activity, using in vitro and cellular-based assays, in whey protein hydrolysates generated using Debitrase and FlavorPro Whey under ST and FF conditions. This appears to be the first report of the influence of enzymatic hydrolysis conditions on cellular antioxidant activity. The higher extent of hydrolysis in the DBT–WPHs may have contributed to more potent in vitro and cellular antioxidant properties when compared with the FPW–WPHs. The WPHs generated under ST conditions exerted stronger TEAC and ORAC activity. However, the antioxidant activity in the HepG2 cell-based assay was not influenced by the hydrolysis conditions used. This is despite the fact that differences in the peptides identified in the WPHs showed that hydrolysis conditions affected enzyme cleavage specificity. 

Our findings extend the results of the previous studies by Le Maux et al. [12], Fernández and Kelly [23] and Butré et al. [26] showing the impact of hydrolysis conditions of whey proteins on the in vitro antioxidant activity, physicochemical properties and peptide profiles. The findings are relevant for the generation of whey protein derived antioxidant peptides at an industrial scale given that the hydrolysis conditions did not affect cellular antioxidant potencies. Nonetheless, the in vivo stability and bioavailability of the WPH-derived peptides remains to be established. Further investigations on the cellular antioxidant properties, specifically on the enzymes involved in oxidative stress as well as the immunomodulatory effects associated with various metabolic syndrome conditions, involving in vitro and in vivo studies are warranted.

## Figures and Tables

**Table 1:** Selected β-lactoglobulin (β-lg)-derived peptides identified in the 4 h whey protein hydrolysates (WPHs) generated using FlavorPro Whey (FPW) and Debitrase (DBT) under pH- and non pH-controlled conditions (ST and FF, respectively) and their bioactive properties as previously reported in the literature (including related peptide sequences).

Peptide Sequence ^1^	Region in Mature β-lg	Identified In				Reported Activity ^2^	IC_50_/EC_50_ Values ^3^	Reference	References to Related Peptide Sequence ^1,2,3,4^
		FPW_ FF	FPW_ ST	DBT_ FF	DBT_ ST				
**KVAGTWYSL**	f(14−22)			✓	✓	-	-	-	VAGTWY (ORAC 5.63 µmol TE/µmol peptide, DPP-IVi IC_50_ 74.9 µM [42], ACEi IC_50_ 1,682 µM [59]), VAGT (ACEi IC_50_ 610.3 µM; ORAC 1.66 µmol TE/mmol peptide [60]), WYSL (DPPH & superoxide scavenging activity with EC_50_ 273.63 & 558.42 µM, respectively [61])
**VAGTW**	f(15−19)				✓	ACEi	534	Pihlanto-LeppÄLÄ, et al. [62]	
**GTWYSL**	f(17−22)			✓		FRAP	na	Bamdad, et al. [9]	
**AMAASDISLL**	f(23−32)			✓		FRAP	na	Bamdad, et al. [9]	AASDISLLDAQSAPLR (antibacterial [63]) MAA (ORAC EC_50_ 0.33 µmol TE/mmol peptide, ACEi IC_50_ 515.5 µM [60])
**MAASDISLL**	f(24−32)			✓		FRAP	na	Bamdad, et al. [9]	
**LDAQSAPLR**	f(32−40)	✓	✓			ACEi	635	Pihlanto-LeppÄLÄ, et al. [62]	
**DAQSAPLRVY**	f(33−42)			✓	✓	ACEi	13	Tavares, et al. [64]	
**DAQSAPLR**	f(33−40)			✓	✓	-	-	-	
**SAPLR**	f(36−40)				✓	-	-	-	
**ELKPTPEGDLEIL**	f(45−57)			✓	✓	FRAP	na	Bamdad, et al. [9]	-
**LKPTPEGDLEIL**	f(46−57)				✓	DPP-IVi	57	Lacroix and Li-Chan [65]	-
**IIAEKTKIPAVF**	f(71−82)			✓	✓	FRAP	na	Bamdad, et al. [9]	IIAEK (ORAC 0.016 µmol TE/µmol peptide, ACEi IC_50_ 63.7 µM [42]), IAEKTKIP (ORAC [10]), IPAVFK (ACEi IC_50_ 144.8 µM, DPP-IVi IC_50_ 149.1 µM, ORAC EC_50_ 0.002 µmol TE/µmol peptide [42])
**AEKTKIPAVF**	f(73−82)	✓		✓	✓	-	-	-	
**AEKTKIPA**	f(73−80)	✓	✓	✓		-	-	-	
**KIPAVF**	f(77−82)				✓	-	-	-	
**IPAVF**	f(78–82)			✓		DPP-IV	44.7	Silveira, et al. [66]	
**VLDTDYKKY**	f(94−102)			✓	✓	-	-	-	LDTDYKKYLLFCMENS (ABTS [67]), DTDYK (ABTS [56]), VLVLDTDYK (DPP-IVi IC_50_ 424.4 µM [66])
**VLDTDYK**	f(94−100)				✓	ABTS	na	Athira, et al. [56]	
						ACEi	946	Pihlanto-LeppÄLÄ, et al. [62]	
**LDTDYKKY**	f(95−102)			✓	✓	-	-	-	
**LDTDYKK**	f(95−101)			✓		ORAC, ACEi	na	Contreras, et al. [10]	
**DTDYKKYLLF**	f(96−105)			✓	✓	-	-	-	
**DTDYKK**	f(96−101)			✓	✓	-	-	-	
**LVRTPEVDDEALEKF**	f(123−135)			✓	✓	-	-	-	VRTPEVDDEALE, LVRTPEVDDEALE, RTPEVDDEALE (ABTS [56])
**VRTPEVDDE**	f(123−131)			✓	✓	ABTS	na	Athira, et al. [56]	
**VRTPEVDDEALEK**	f(123−134)			✓	✓	-	-	-	
**RTPEVDDEALEK**	f(124−134)			✓	✓	-	-	-	
**TPEVDDEALEK**	f(125−135)	✓	✓	✓	✓	DPP-IVi	319.5	Silveira, et al. [66]	
						ORAC	0.004	Power, et al. [42]	
						ABTS	na	Mann, et al. [57]	
**ALKALPM**	f(139−145)				✓	-	-	-	ALPMHIR (ACEi IC_50_ 43 µM [59], ORAC EC_50_ 0.035 µmol TE/µmol peptide [42])
**KALPM**	f(141−145)				✓	-	-	-	
**ALPMH**	f(142−146)		✓	✓	✓	ACEi	521	Mullally, et al. [59]	
						DPP-IVi	>100	Tulipano, et al. [68]	

^1^ Peptide sequences presented with the one letter code. ^2^ ACEi: angiotensin I-converting enzyme inhibitory activity; DPP-IVi: dipeptidyl peptidase IV inhibitory activity; FRAP: ferric reducing antioxidant power; ORAC: oxygen radical antioxidant capacity. ^3^ IC_50_: concentration of peptide resulting in 50% inhibition of ACE and DPP-IV activity reported as µM peptide; EC_50_: half maximal effective concentration of peptide reported as µmol Trolox equivalent/µmol peptide; na: not applicable (no reported value). ^4^ DPPH: 2,2-diphenyl-1-picrylhydrazyl scavenging activity; ABTS: 2,2’-azino-bis-(3-ethylbenzothiazoline)-6-sulfonic acid radical scavenging activity.

## Supplementary Materials

Supplementary data associated with this manuscript are available online at https://www.mdpi.com/2304-8158/9/5/582/s1, including **Figure S1:** Effect of different concentrations of oxidative stress inducers, 2,2’-azobis(2-amidinopropane) dihydrochloride (AAPH) and hydrogen peroxide (H_2_O_2_), on HepG2 cells viability. The results were expressed as the percentage of viable cells remaining following treatment with oxidative stress inducers compared to untreated control cells. Values represent mean ± SD (*n* = 3), **Figure S2:** Viability of HepG2 cells treated with the 4 h whey protein hydrolysates (WPHs) generated using FlavorPro Whey (FPW) and Debitrase (DBT) under pH-(ST) and non pH-controlled (FF) conditions. The results were expressed as the percentage of viable cells remaining following treatment with WPHs compared to untreated control cells. Values represent mean ± SD (*n* = 3).

## References

[1] Brandelli A., Daroit D.J., Corrêa A.P.F. (2015). Whey as a source of peptides with remarkable biological activities. Food Res. Int..

[2] Nongonierma A.B., O’Keeffe M.B., FitzGerald R.J., McSweeney P.L.H., O’Mahony J.A. (2016). Milk protein hydrolysates and bioactive peptides. Advanced Dairy Chemistry: Volume 1b: Proteins: Applied Aspects.

[3] Corrochano A.R., Buckin V., Kelly P.M., Giblin L. (2018). Invited review: Whey proteins as antioxidants and promoters of cellular antioxidant pathways. J. Dairy Sci..

[4] Khan I.T., Nadeem M., Imran M., Ullah R., Ajmal M., Jaspal M.H. (2019). Antioxidant properties of milk and dairy products: A comprehensive review of the current knowledge. Lipids Health Dis..

[5] Mangano K.M., Bao Y., Zhao C., Guo M. (2019). Nutritional properties of whey proteins. Whey Protein Production, Chemistry, Functionality, and Applications.

[6] Poprac P., Jomova K., Simunkova M., Kollar V., Rhodes C.J., Valko M. (2017). Targeting free radicals in oxidative stress-related human diseases. Trends Pharm. Sci..

[7] Niki E. (2010). Assessment of antioxidant capacity in vitro and in vivo. Free Radic. Biol. Med..

[8] Udenigwe C.C., Aluko R.E. (2012). Food protein-derived bioactive peptides: Production, processing, and potential health benefits. J. Food Sci..

[9] Bamdad F., Bark S., Kwon C.H., Suh J.-W., Sunwoo H. (2017). Anti-inflammatory and antioxidant properties of peptides released from β-lactoglobulin by high hydrostatic pressure-assisted enzymatic hydrolysis. Molecules.

[10] Contreras M.d.M., Hernández-Ledesma B., Amigo L., Martín-Álvarez P.J., Recio I. (2011). Production of antioxidant hydrolyzates from a whey protein concentrate with thermolysin: Optimization by response surface methodology. LWT Food Sci. Technol..

[11] Jiang Z., Yao K., Yuan X., Mu Z., Gao Z., Hou J., Jiang L. (2018). Effects of ultrasound treatment on physicochemical, functional properties and antioxidant activity of whey protein isolate in the presence of calcium lactate. J. Sci. Food Agric..

[12] Le Maux S., Nongonierma A.B., Barre C., FitzGerald R.J. (2016). Enzymatic generation of whey protein hydrolysates under pH-controlled and non pH-controlled conditions: Impact on physicochemical and bioactive properties. Food Chem..

[13] Madadlou A., Abbaspourrad A. (2018). Bioactive whey peptide particles: An emerging class of nutraceutical carriers. Crit. Rev. Food Sci. Nutr..

[14] Neto Y.A.A.H., Rosa J.C., Cabral H. (2019). Peptides with antioxidant properties identified from casein, whey, and egg albumin hydrolysates generated by two novel fungal proteases. Prep. Biochem. Biotechnol..

[15] O’Keeffe M.B., FitzGerald R.J. (2014). Antioxidant effects of enzymatic hydrolysates of whey protein concentrate on cultured human endothelial cells. Int. Dairy J..

[16] Power O., Jakeman P., FitzGerald R.J. (2013). Antioxidative peptides: Enzymatic production, in vitro and in vivo antioxidant activity and potential applications of milk-derived antioxidative peptides. Amino Acids.

[17] Salami M., Moosavi-Movahedi A.A., Ehsani M.R., Yousefi R., Haertlé T., Chobert J.-M., Razavi S.H., Henrich R., Balalaie S., Ebadi S.A. (2010). Improvement of the antimicrobial and antioxidant activities of camel and bovine whey proteins by limited proteolysis. J. Agric. Food Chem..

[18] Korhonen H., Pihlanto A. (2006). Bioactive peptides: Production and functionality. Int. Dairy J..

[19] Tagliazucchi D., Helal A., Verzelloni E., Conte A. (2016). Bovine milk antioxidant properties: Effect of in vitro digestion and identification of antioxidant compounds. Dairy Sci. Technol..

[20] De Carvalho N.C., Pessato T.B., Fernandes L.G.R., De Lima Zollner R., Netto F.M. (2017). Physicochemical characteristics and antigenicity of whey protein hydrolysates obtained with and without pH control. Int. Dairy J..

[21] Cheison S.C., Kulozik U. (2017). Impact of the environmental conditions and substrate pre-treatment on whey protein hydrolysis: A review. Crit. Rev. Food Sci. Nutr..

[22] Cheison S.C., Leeb E., Toro-Sierra J., Kulozik U. (2011). Influence of hydrolysis temperature and pH on the selective hydrolysis of whey proteins by trypsin and potential recovery of native alpha-lactalbumin. Int. Dairy J..

[23] Fernández A., Kelly P. (2016). pH-stat vs. Free-fall pH techniques in the enzymatic hydrolysis of whey proteins. Food Chem..

[24] Butré C.I., Sforza S., Wierenga P.A., Gruppen H. (2015). Determination of the influence of the pH of hydrolysis on enzyme selectivity of *Bacillus licheniformis* protease towards whey protein isolate. Int. Dairy J..

[25] Butré C.I., Wierenga P.A., Gruppen H. (2012). Effects of ionic strength on the enzymatic hydrolysis of diluted and concentrated whey protein isolate. J. Agric. Food Chem..

[26] Butré C.I., Sforza S., Gruppen H., Wierenga P.A. (2014). Introducing enzyme selectivity: A quantitative parameter to describe enzymatic protein hydrolysis. Anal. Bioanal. Chem..

[27] Apak R., Özyürek M., Güçlü K., Çapanoğlu E. (2016). Antioxidant activity/capacity measurement. 2. Hydrogen atom transfer (HAT)-based, mixed-mode (electron transfer (ET)/HAT), and lipid peroxidation assays. J. Agric. Food Chem..

[28] Spellman D., McEvoy E., O’Cuinn G., FitzGerald R.J. (2003). Proteinase and exopeptidase hydrolysis of whey protein: Comparison of the TNBS, OPA and pH stat methods for quantification of degree of hydrolysis. Int. Dairy. J..

[29] O’Loughlin I.B., Murray B.A., Kelly P.M., FitzGerald R.J., Brodkorb A. (2012). Enzymatic hydrolysis of heat-induced aggregates of whey protein isolate. J. Agric. Food Chem..

[30] Spellman D., O’Cuinn G., FitzGerald R.J. (2009). Bitterness in *Bacillus* proteinase hydrolysates of whey proteins. Food Chem..

[31] Le Maux S., Nongonierma A.B., Lardeux C., FitzGerald R.J. (2018). Impact of enzyme inactivation conditions during the generation of whey protein hydrolysates on their physicochemical and bioactive properties. Int. J. Food Sci. Technol..

[32] Re R., Pellegrini N., Proteggente A., Pannala A., Yang M., Rice-Evans C. (1999). Antioxidant activity applying an improved ABTS radical cation decolorization assay. Free Radic. Biol. Med..

[33] Yarnpakdee S., Benjakul S., Kristinsson H.G., Bakken H.E. (2015). Preventive effect of nile tilapia hydrolysate against oxidative damage of HepG2 cells and DNA mediated by H_2_O_2_ and AAPH. J. Food Sci. Technol..

[34] Nongonierma A.B., Cadamuro C., Le Gouic A., Mudgil P., Maqsood S., FitzGerald R.J. (2019). Dipeptidyl peptidase IV (DPP-IV) inhibitory properties of a camel whey protein enriched hydrolysate preparation. Food Chem..

[35] O’Keeffe M.B., FitzGerald R.J. (2015). Identification of short peptide sequences in complex milk protein hydrolysates. Food Chem..

[36] Heberle H., Meirelles G.V., Da Silva F.R., Telles G.P., Minghim R. (2015). InteractiVenn: A web-based tool for the analysis of sets through Venn diagrams. BMC Bioinform..

[37] Haileselassie S.S., Lee B.H., Gibbs B.F. (1999). Purification and identification of potentially bioactive peptides from enzyme-modified cheese. J. Dairy Sci..

[38] Nongonierma A.B., FitzGerald R.J., Fuquay J.W. (2011). Enzymes exogenous to milk in dairy technology | Proteinases. Encyclopedia of Dairy Sciences.

[39] Spellman D., O’Cuinn G., FitzGerald R.J. (2005). Physicochemical and sensory characteristics of whey protein hydrolysates generated at different total solids levels. J. Dairy Res..

[40] O’Sullivan D., Nongonierma A.B., FitzGerald R.J. (2017). Bitterness in sodium caseinate hydrolysates: Role of enzyme preparation and degree of hydrolysis. J. Sci. Food Agric..

[41] Kalyankar P., Zhu Y., O’Cuinn G., FitzGerald R.J. (2013). Investigation of the substrate specificity of glutamyl endopeptidase using purified bovine β-casein and synthetic peptides. J. Agric. Food Chem..

[42] Power O., Fernández A., Norris R., Riera F.A., FitzGerald R.J. (2014). Selective enrichment of bioactive properties during ultrafiltration of a tryptic digest of β-lactoglobulin. J. Funct. Foods.

[43] Giblin L., Yalçın A.S., Biçim G., Krämer A.C., Chen Z., Callanan M.J., Arranz E., Davies M.J. (2019). Whey proteins: Targets of oxidation, or mediators of redox protection. Free Radic. Res..

[44] Xu R., Liu N., Xu X., Kong B. (2011). Antioxidative effects of whey protein on peroxide-induced cytotoxicity. J Dairy Sci..

[45] Corrochano A.R., Ferraretto A., Arranz E., Stuknytė M., Bottani M., O’Connor P.M., Kelly P.M., De Noni I., Buckin V., Giblin L. (2019). Bovine whey peptides transit the intestinal barrier to reduce oxidative stress in muscle cells. Food Chem..

[46] Kong B., Peng X., Xiong Y.L., Zhao X. (2012). Protection of lung fibroblast MRC-5 cells against hydrogen peroxide-induced oxidative damage by 0.1–2.8 kDa antioxidative peptides isolated from whey protein hydrolysate. Food Chem..

[47] Zhang Q.-X., Ling Y.-F., Sun Z., Zhang L., Yu H.-X., Kamau S.M., Lu R.-R. (2012). Protective effect of whey protein hydrolysates against hydrogen peroxide-induced oxidative stress on PC12 cells. Biotechnol. Lett..

[48] Piccolomini A., Iskandar M., Lands L., Kubow S. (2012). High hydrostatic pressure pre-treatment of whey proteins enhances whey protein hydrolysate inhibition of oxidative stress and IL-8 secretion in intestinal epithelial cells. Food Nutr. Res..

[49] Iskandar M., Lands L., Sabally K., Azadi B., Meehan B., Mawji N., Skinner C., Kubow S. (2015). High hydrostatic pressure pretreatment of whey protein isolates improves their digestibility and antioxidant capacity. Foods.

[50] Pyo M.C., Yang S.-Y., Chun S.-H., Oh N.S., Lee K.-W. (2016). Protective effects of maillard reaction products of whey protein concentrate against oxidative stress through an Nrf2-dependent pathway in HepG2 cells. Biol. Pharm. Bull..

[51] Kim G.-N., Kwon Y.-I., Jang H.-D. (2011). Protective mechanism of quercetin and rutin on 2,2′-azobis(2-amidinopropane)dihydrochloride or Cu^2+^-induced oxidative stress in HepG2 cells. Toxicol. In Vitro.

[52] Honzel D., Carter S.G., Redman K.A., Schauss A.G., Endres J.R., Jensen G.S. (2008). Comparison of chemical and cell-based antioxidant methods for evaluation of foods and natural products: Generating multifaceted data by parallel testing using erythrocytes and polymorphonuclear cells. J. Agric. Food Chem..

[53] Lima C.F., Fernandes-Ferreira M., Pereira-Wilson C. (2006). Phenolic compounds protect HepG2 cells from oxidative damage: Relevance of glutathione levels. Life Sci..

[54] Athira S., Mann B., Sharma R., Kumar R. (2013). Ameliorative potential of whey protein hydrolysate against paracetamol-induced oxidative stress. J. Dairy Sci..

[55] Ashok N.R., Vivek K.H., Aparna H.S. (2019). Antioxidative role of buffalo (*Bubalus bubalis*) colostrum whey derived peptides during oxidative damage. Int. J. Pept. Res..

[56] Athira S., Mann B., Saini P., Sharma R., Kumar R., Singh A.K. (2015). Production and characterisation of whey protein hydrolysate having antioxidant activity from cheese whey. J. Sci. Food Agric..

[57] Mann B., Kumari A., Kumar R., Sharma R., Prajapati K., Mahboob S., Athira S. (2015). Antioxidant activity of whey protein hydrolysates in milk beverage system. J. Food Sci. Technol..

[58] Picariello G., Iacomino G., Mamone G., Ferranti P., Fierro O., Gianfrani C., Di Luccia A., Addeo F. (2013). Transport across Caco-2 monolayers of peptides arising from *in vitr*o digestion of bovine milk proteins. Food Chem..

[59] Mullally M.M., Meisel H., FitzGerald R.J. (1997). Identification of a novel angiotensin-I-converting enzyme inhibitory peptide corresponding to a tryptic fragment of bovine β-lactoglobulin. FEBS Lett..

[60] O’Keeffe M.B., Conesa C., FitzGerald R.J. (2017). Identification of angiotensin converting enzyme inhibitory and antioxidant peptides in a whey protein concentrate hydrolysate produced at semi-pilot scale. Int. J. Food Sci. Technol..

[61] Hernández-Ledesma B., Miguel M., Amigo L., Aleixandre M.A., Recio I. (2007). Effect of simulated gastrointestinal digestion on the antihypertensive properties of synthetic β-lactoglobulin peptide sequences. J. Dairy Res..

[62] Pihlanto-LeppÄLÄ A., Koskinen P., Piilola K., Tupasela T., Korhonen H. (2000). Angiotensin I-converting enzyme inhibitory properties of whey protein digests: Concentration and characterization of active peptides. J. Dairy Res..

[63] Pellegrini A., Dettling C., Thomas U., Hunziker P. (2001). Isolation and characterization of four bactericidal domains in the bovine β-lactoglobulin. Biochim. Biophys. Acta (BBA) Gen. Subj..

[64] Tavares T., Contreras M.d.M., Amorim M., Pintado M., Recio I., Malcata F.X. (2011). Novel whey-derived peptides with inhibitory effect against angiotensin-converting enzyme: In vitro effect and stability to gastrointestinal enzymes. Peptides.

[65] Lacroix I.M.E., Li-Chan E.C.Y. (2014). Overview of food products and dietary constituents with antidiabetic properties and their putative mechanisms of action: A natural approach to complement pharmacotherapy in the management of diabetes. Mol. Nutr. Food Res..

[66] Silveira H., Moraes H., Oliveira N., Coutinho E.S.F., Laks J., Deslandes A. (2013). Physical exercise and clinically depressed patients: A systematic review and meta-analysis. Neuropsychobiology.

[67] Bertucci J.I., Liggieri C.S., Colombo M.L., Vairo Cavalli S.E., Bruno M.A. (2015). Application of peptidases from *Maclura pomifera* fruit for the production of active biopeptides from whey protein. LWT Food Sci. Technol..

[68] Tulipano G., Sibilia V., Caroli A.M., Cocchi D. (2011). Whey proteins as source of dipeptidyl dipeptidase IV (dipeptidyl peptidase-4) inhibitors. Peptides.

[69] Tulipano G., Faggi L., Nardone A., Cocchi D., Caroli A.M. (2015). Characterisation of the potential of β-lactoglobulin and α-lactalbumin as sources of bioactive peptides affecting incretin function: *In silico* and in vitro comparative studies. Int. Dairy J..

[70] FitzGerald R., Meisel H. (1999). Lactokinins: Whey protein-derived ACE inhibitory peptides. Food/Nahrung.

[71] Maes W., Van Camp J., Vermeirssen V., Hemeryck M., Ketelslegers J.M., Schrezenmeir J., Van Oostveldt P., Huyghebaert A. (2004). Influence of the lactokinin Ala-Leu-Pro-Met-His-Ile-Arg (ALPMHIR) on the release of endothelin-1 by endothelial cells. Regul. Pept..

