# Supplementary material for: In Vitro Characterisation of the Antioxidative Properties of Whey Protein Hydrolysates Generated under pH- and Non pH-Controlled Conditions
Source: Foods. 2020 May 5;9(5):582. doi: 10.3390/foods9050582 (PMC7278780; doi:10.3390/foods9050582)
Supplement: Supplementary file 1 [file foods-09-00582-s001.pdf]

foods

MDPI

Supplementary data

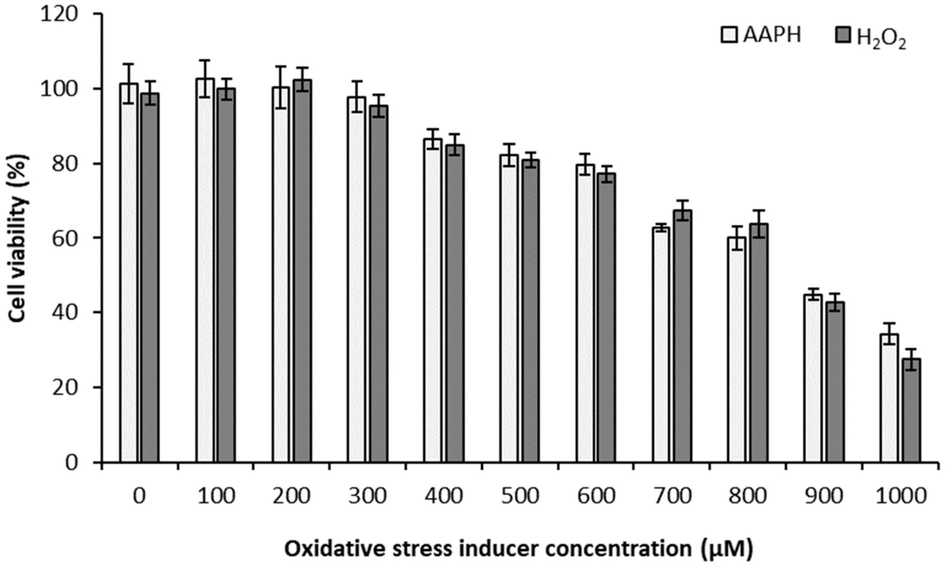

Figure S1 Effect of different concentrations of oxidative stress inducers, 2,2'-azobis(2-amidinopropane) dihydrochloride (AAPH) and hydrogen peroxide (H_2O_2), on HepG2 cell viability. The results were expressed as the percentage of viable cells remaining following treatment with oxidative stress inducers compared to untreated control cells. Values represent mean ± SD (*n*=3).

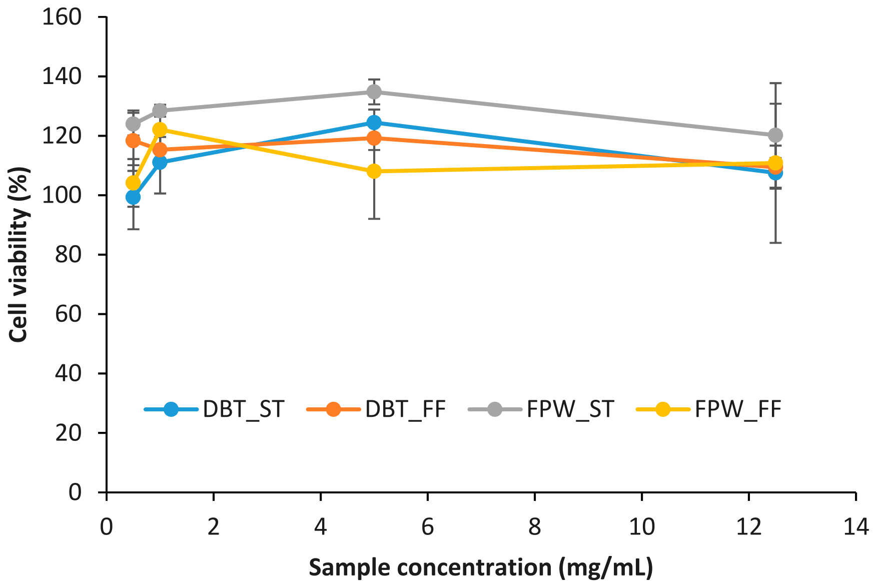

Figure S2 Viability of HepG2 cells treated with the 4 h whey protein hydrolysates (WPHs) generated using FlavorPro Whey (FPW) and Debitrase (DBT) under pH- (ST) and non pH-controlled (FF) conditions. The results were expressed as the percentage of viable cells remaining following treatment with the different WPHs compared to untreated control cells. Values represent mean ± SD (*n*=3).
